# Serological assessment of gastric mucosal atrophy in gastric cancer

**DOI:** 10.1186/1471-230X-12-10

**Published:** 2012-01-31

**Authors:** Jan Bornschein, Michael Selgrad, Thomas Wex, Doerthe Kuester, Peter Malfertheiner

**Affiliations:** 1 Department of Gastroenterology, Hepatology and Infectious Diseases, Otto-von-Guericke-University of Magdeburg, Leipziger Str. 44, D-39120 Magdeburg, Germany; 2 Department of Pathology, Otto-von-Guericke-University of Magdeburg, Leipziger Str. 44, D-39120 Magdeburg, Germany

**Keywords:** Gastric cancer, *Helicobacter pylori*, intestinal metaplasia, glandular atrophy, gastrin, pepsinogen, cardia cancer

## Abstract

**Background:**

Non-invasive tools for gastric cancer screening and diagnosis are lacking. Serological testing with the detection of pepsinogen 1 (PG1), pepsinogen 2 (PG2) and gastrin 17 (G17) offers the possibility to detect preneoplastic gastric mucosal conditions. Aim of this study was to assess the performance of these serological tests in the presence of gastric neoplasia.

**Methods:**

Histological and serological samples of 118 patients with gastric cancer have been assessed for tumor specific characteristics (Laurén type, localisation), degree of mucosal abnormalities (intestinal metaplasia, atrophy) and serological parameters (PG1, PG2, PG1/2-ratio, G17, *H. pylori *IgG, CagA status). Association of the general factors to the different serological values have been statistically analyzed.

**Results:**

Patients with intestinal type gastric cancer had lower PG1 levels and a lower PG1/2-ratio compared to those with diffuse type cancer (*p *= 0.003). The serum levels of PG2 itself and G17 were not significantly altered. *H. pylori *infection in general had no influence on the levels of PG1, PG2 and G17 in the serum of gastric cancer patients. There was a trend towards lower PG1 levels in case of positive CagA-status (*p *= 0.058). The degree of both intestinal metaplasia and atrophy correlated inversely with serum levels for PG1 and the PG1/2-ratio (p < 0.01). Laurén-specific analysis revealed that this is only true for intestinal type tumors. Univariate ANOVA revealed atrophy and CagA-status as the only independent factors for low PG1 and a low PG1/2-ratio.

**Conclusions:**

Glandular atrophy and a positive CagA status are determinant factors for decreased pepsinogen 1 levels in the serum of patients with gastric cancer. The serological assessment of gastric atrophy by analysis of serum pepsinogen is only adequate for patients with intestinal type cancer.

## Background

Most of the patients report only a short period of symptoms appearing before the establishment of the first diagnosis of gastric cancer (GC). Up to 40% report not to have any dyspeptic symptoms at all [1]. The prognosis is dismal in most cases, and therefore, an adequate and cost-effective screening program to enable early detection of the disease is needed to reduce gastric cancer-related mortality [2]. Population mass screening for GC has only been conducted in high incidence regions in Asia with good results by lowering the mortality from GC in Korea and Japan [3,4].

Endoscopy with sampling of gastric biopsies was documented as the best and most effective option for screening for upper GI malignancies [4]. Based on retrospective data from Singapore it has been estimated that endoscopic screening for stomach cancer can be cost-effective only in moderate to high-risk populations [5]. Thus, endoscopic screening is not applicable in low risk regions and therefore non-invasive screening modalities are needed in these populations. In the absence of reliable biomarkers for the detection of gastric cancer, a screening program would include the evaluation of surrogate markers such as the detection of *Helicobacter pylori (H. pylori)*, and the serological characterization of preneoplastic conditions of the gastric mucosa.

This concept fits best to the intestinal type of GC with the well described progression from *H. pylori *driven chronic gastritis via atrophic gastritis, intestinal metaplasia (IM) and intraepithelial neoplasia (formerly called dysplasia) to invasive gastric cancer [6]. At a lower prevalence, gastric atrophy and IM are also reported in association with diffuse type carcinomas [7]. Glandular atrophy in the body can be regarded as premalignant condition [8], and the risk for gastric carcinogenesis has been reported to be increased and correlated with the degree of baseline atrophy [9]. For non-invasive detection and grading of gastric atrophy pepsinogen I (PG1), pepsinogen II (PG2), and gastrin 17 (G17) in the serum are suitable parameters [10-12].

In a meta-analysis evaluating more than 40 studies with about 300,000 individuals included, Miki and colleagues reported that tests on serum pepsinogens are not appropriate for GC screening but may be useful for identification of high-risk individuals who necessitate further diagnostic work-up [13]. These conclusions were confirmed by recent studies [14-17].

The serological analysis for *H. pylori*-infection should be included in further analyses [18], since the presence of *H. pylori *can increase the risk for gastric carcinogenesis independently from the presence of mucosal atrophy [16,17].

So far, there was no success to include the available data in the algorithms for serological gastric cancer screening. The aim of the present study was to evaluate the association of specific gastric cancer characteristics and the *H. pylori *status in combination with the serum levels of PG1, PG2 and G17. Since it was not aim of the study to assess the diagnostic value of these tests general but the confounding influence of gastric cancer we did not include the evaluation of a healthy control group.

## Methods

Patients with GC were enrolled in the course of a different study that was approved by the local ethic committee. Written informed consent was obtained. The available material was re-evaluated for the presence of *H. pylori *infection, including CagA status, and histopathological mucosal alterations. The groups were analyzed according to their histopathological type of carcinoma (i.e. intestinal vs. diffuse type by Laurén) and the location of the primary tumor. Serum samples were assessed for the content of gastrin 17 (G17), pepsinogen 1 (PG1) and pepsinogen 2 (PG2). These analyses have been performed in concordance with the guidelines of the local ethics committee.

### Data assessment

Patients' data was verified using the electronic patient documentation system of the Department of Gastroenterology, Hepatology and Infectious Diseases at the University Hospital of Magdeburg, Germany. Patients were included in case of complete records and availability of adequately stored serum samples for serological analysis (-80°C). In total, 118 patients met these criteria.

Only patients with either intestinal (n = 59) or diffuse type tumors (n = 59) according to the Laurén classification were included for further analysis, mixed type tumors were excluded (n = 12). Exclusion of mixed type tumors was done to analyse only clearly distinct tumor entities. Since "Laurén type" was considered as a potential confounding factor for the serological tests, we aimed for a strict comparison between intestinal and diffuse type gastric cancer.

Tumors were classified according to the location of the main tumor mass into carcinomas of the antrum, the corpus and the cardia. Cardia tumors were further subclassified according to the AEG-classification as proposed by Siewert and Stein in 1998 (AEG = adenocarcinoma of the esophagogastric junction) [19]. Supracardial carcinomas (AEG1) with a high likelihood of the distal esophagus being the site of origin have been excluded, as well as AEG2 with adjacent Barrett's mucosa. Tumors have been stratified into "proximal" and "distal" carcinomas of the stomach according to the location of the main tumor mass as described previously [7]. Tumors in the region of the esophagogastric junction were clearly classified as "proximal" and antrum-tumors as "distal". The group of corpus-carcinomas was subdivided. Carcinomas with the main tumor mass in the fundus and in the proximal third of the corpus were classified as "proximal", all more distally located corpus carcinomas (lower two thirds) as "distal".

Tumor allocation was assessed either by the primary endoscopy report including endoscopic ultrasound or by the pathological report of the resected specimen in case of gastrectomy. This classification of tumor allocation was chosen by convention of the authors. Tumor allocation was stratified prospectively to any statistical analysis.

### Histopathological assessment

Histopathological alterations of the gastric mucosa (IM and glandular atrophy) were assessed according to the updated Sydney system [20].

Biopsies had been taken in duplicate from the pre-pyloric antrum and the corpus (lesser and greater curvature). Cardia-derived samples were obtained directly below the Z-Line at the proximal end of the gastric folds. Additional samples were taken from the tumor itself and in some cases from the surrounding mucosa.

Biopsies were processed by routine methods. One section was stained with hematoxilin and eosin, modified Giemsa for detection of *H. pylori*, and PAS stain.

In patients that were referred to our hospital for gastrectomy with no further endoscopic examination being performed, histology was assessed on the surgical specimen.

### Assessment of serum parameters

Serum was prepared from 5-7 ml venous blood by centrifugation at 7.000x g at 4°C for 15 min, aliquoted in three individual cryotubes (each 1-1.5 ml) within 3 hours after taking blood. Samples were stored at -80°C until analysis. The concentration of anti-*H. pylori *IgG antibodies were analyzed using the Pyloriset EIA-G III (Orion Diagnostica, Finland) according to the manufacturer's instructions. According to the validation of the kit, a positive result was defined as ≥ 30 EIU, a negative result as < 30 EIU. The prevalence of anti-CagA antibodies was investigated using undiluted sera and the *Helicobacter pylori *Vira blot test kit (Viramed Biotech AG, Lich, Germany) according to the instructions by the manufacturer. Patients with positive CagA-status were classified as "*H. pylori *positive" even when anti-*H. pylori *IgG was below the cut-off level also including patients with "a serological scar". It has to be mentioned that by this definition not only patients with actual infection were classified as *H. pylori*-positive, but also patients with prior eradication therapy or those in which bacteria have disappeared during the progression of histological alterations. Since the so-called "point of no return" from which on eradication cannot prevent further progression of premalignant conditions is not defined yet, we believe that inclusion of all patients with a past history of *H. pylori *infection is justified.

The analyses of anti-*H. pylori *IgG antibodies and PG1, PG2 and G17 (Gastrin-17 EIA Test Kit, Pepsinogen-I, Pepsinogen-II EIA Test Kit, Biohit Plc, Finland) was performed at the same aliquot and as described by the manufacturer.

### Statistical analysis

Group comparison was performed using Fisher's exact test, correlation analysis by Spearman's rank correlation test. The t-test for independent samples was applied to assess the influence of age as a confounding factor and to compare the degree of mucosal alterations between groups. Group comparison concerning the serum values of G17, PG1 and PG2, as well as the PG-ratio have been performed by the Mann-Whitney U-test as well as univariate ANOVA for interference analysis. For all tests, a two-sided significance level of *P *< 0.05 was assumed. Data were analyzed using SPSS 11.0 (SPSS Inc., Chicago, IL, USA).

## Results

### General characteristics of the study population

The general characteristics of the study population are described in table 1.

**Table 1:** General characteristics of the study population

		Intestinal GC (n = 59)		Diffuse GC (n = 59)		Total (n = 118)	
Age*	(mean ± SD)	67.5 ± 12.5	years	62.2 ± 13.6	years	64.9 ± 13.3	years
Sex*	(female)	42	71.2%	30	50.8%	72	61.0%

*H. pylori*	(positive)	50	84.7%	49	83.1%	99	83.9%
CagA	(positive in total)	39	66.1%	38	64.4%	77	65.3%
	(of H.pylori-positive)		78.0%		77.6%		77.8%

Localisation*	Cardia	21	35.6%	13	22.0%	34	28.8%
	Corpus	21	35.6%	37	62.7%	58	49.2%
	Antrum	17	28.8%	9	15.3%	26	22.0%
	
	Proximal	30	50.8%	23	39%	53	44.9%
	Distal	29	49.2%	36	61%	65	55.1%

Atrophy*	(positive)	33	55.9%	10	16.9%	43	36.4%
IM*	(positive)	51	86.4%	35	59.3%	86	72.9%

PG1	(median, range)	81.9 (4.2-699.8) ng/ml		127.9 (5.3-877.5) ng/ml		103.3 (4.2-877.5) ng/ml	
PG2	(median, range)	13.9 (3.2-173.5) ng/ml		13.1 (2.5-73.1) ng/ml		13.5 (2.5-173.5) ng/ml	
PG-ratio*	(median, range)	6.8 (0.4-25.8)		10.4 (1.6-26.7)		8.3 (0.4-26.7)	
G17	(median, range)	13.8 (0.1-220.0) pM		12.8 (0.6-194.2) pM		13.4 (0.1-220.0) pM	

Serum parameters are illustrated by median and range. Patients were regarded as "positive" for atrophy and IM if the Sydney-scaled degree of the mucosal alteration was ≥ 1. Significant differences between intestinal and diffuse type GC are marked with an asterisk (*) applying a two-sided significance level of *p *< 0.05.

Patients with intestinal type tumors were significantly younger than those with diffuse type GC (*p *= 0.029), and showed a higher proportion of female patients (*p *= 0.037). There was no significant difference in the distribution of proximal and distal GC between the two Laurén types. However, there were more corpus carcinomas in the group with diffuse type GC (*p *= 0.013). Both *H. pylori *and CagA status were similar between the two Laurén types. There was a higher prevalence for IM and glandular atrophy in case of intestinal type GC compared to diffuse type tumors (*p *= 0.002, *p *< 0.001, resp.). Table 2 gives an overview about for the distribution of mucosal changes in both Laurén types.

**Table 2:** Degree of atrophy and IM according to the updated Sydney-classification

		Intestinal GC (n = 59)	Diffuse GC (n = 59)	Total (n = 118)
Atrophy	Antrum*	0.71 ± 0.83	0.21 ± 0.58	0.47 ± 0.76
	Corpus*	0.64 ± 0.83	0.29 ± 0.65	0.47 ± 0.77
	Total*	0.90 ± 0.87	0.32 ± 0.68	0.61 ± 0.83

IM	Antrum*	1.32 ± 0.83	0.69 ± 0.72	1.01 ± 0.84
	Corpus*	0.91 ± 0.88	0.51 ± 0.73	0.71 ± 0.83
	Total*	1.47 ± 0.82	0.80 ± 0.76	1.14 ± 0.86

Displayed are mean and standard deviation for the Sydney-scaled degree of glandular atrophy and IM for each antrum and corpus (for "total", the highest score of both localisations was counted). Significant differences between intestinal and diffuse type GC are marked with an asterisk (*) applying a two-sided significance level of *p *< 0.05.

### Pepsinogens and G17 in the serum and pathobiological characteristics

Patients with diffuse type GC presented with 1.56-fold higher PG1 levels compared to those with intestinal type tumors (127.9 ng/ml vs. 81.9 ng/ml). This difference was not statistically significant (*p *= 0.062; additional file 1). Values of the PG1/2-ratio were significantly higher in case of diffuse type carcinomas (10.4 vs. 6.8; *p *= 0.003; table 1; additional file 2). There was no difference concerning the serum levels of G17 and PG2 between GC of the intestinal and diffuse type.

Generally, serum levels of PG1, PG2 and G17 were similar between *H. pylori*-positive and -negative subjects. A trend towards lower PG1 serum levels (94.6 ng/ml vs. 141.6 ng/ml; *p *= 0.058) was observed for patients with positive CagA status compared to negative ones (table 1; additional file 1).

There was no difference in serum parameters between carcinomas with proximal and distal location.

### Pepsinogens and G17 in the serum and mucosal changes

Patients with presence of atrophy showed significantly lower levels of PG1 (*p *< 0.001) as well as a lower PG1/2-ratio (*p *> 0.001) than those without atrophy (Table 3; additional file 2). This was confirmed for patients with intestinal type tumors for both PG1 (*p *= 0.003) and the PG1/2-ratio (*p *= 0.028).

**Table 3:** PG1 in the serum and the PG1/2-ratio in association to gastric mucosal alterations

	Intestinal GC (n = 59)		Diffuse GC (n = 59)		Total (n = 118)	
	**PG1 (ng/ml)**	**PG1/2-ratio**	**PG1 (ng/ml)**	**PG1/2-ratio**	**PG1 (ng/ml)**	**PG1/2-ratio**

Atrophy						
(pos. vs. neg.)	50.4 vs. 120.6*	4.4 vs. 8.1*	77.9 vs. 141.6	7.0 vs. 11.0	56.2 vs. 136.6*	5.0 vs. 10.4*

IM						
(pos. vs. neg.)	85.6 vs. 80.2	7.0 vs. 5.6	87.6 vs. 159.5*	8.4 vs. 11.5	86.3 vs. 147.8*	7.5 vs. 10.4*

Displayed are medians of PG1 in the serum and of the PG1/2-ratio for patients with intestinal type and diffuse type GC as well as for the whole study population (range not shown). For each line values for patients with and without atrophy or IM are given in direct comparison. Significant differences are marked with an asterisk applying a two-sided significance level of *p *< 0.05.

Patients with IM in the stomach demonstrated both lower PG1 values (*p *= 0.02) and a lower PG1/2-ratio (*p *= 0.006) than patients without metaplasia (additional file 2). Laurén specific comparison could confirm this result for PG1 levels in case of diffuse type carcinomas (*p *= 0.029).

There was no significant influence of the mucosal changes on the absolute levels of PG2 and G17 in the serum.

Serum levels of PG1 correlated inversely with the degree of gastric glandular atrophy (*p *< 0.001; *r *= -0.385). This relation was also demonstrated for the PG1/2-ratio (*p *= 0.001; *r *= -0.344) (additional file 3). According to the updated Sydney-classification, mucosal changes have been graded independently for antrum and corpus. Location-specific correlation-analyses revealed similar results for patients with atrophic foci in the antrum and the corpus (data not shown).

PG1 (*p *< 0.001, *r *= -0.351) and the PG1/2-ratio (*p *= 0.002; *r *= -0.285) correlated inversely with the degree of IM (additional file 3). Subgroup analysis did not show any difference between IM in antrum and corpus (data not shown).

There was no correlation of absolute serum values for PG2 with the degree of any mucosal alteration. G17 showed an inverse correlation with the degree of IM (not with atrophy) in the antrum (*p *= 0.044; *r *= -0.191; data not shown).

Both the degree of IM and the degree of atrophy correlated with the patients' age (IM: *p *< 0.001, *r *= 0.341; atrophy: *p *= 0.003, *r *= 0.272) (additional file 4).

All parameters mentioned above (Laurén type, localisation, IM, atrophy, *H. pylori *status, CagA status) have been evaluated by univariate ANOVA-analysis. Only the presence of atrophy (*p *= 0.022) and a positive CagA status (*p *= 0.013) had an influence on the serum levels of PG1. For none of the parameters analysed a significant influence on the PG1/2-ratio could be identified. Age was respected in this analysis but did not show an influence.

### Subgroup characteristics in case of pathological pepsinogen test

To analyse the subgroup characteristics of patients with pathological pepsinogen test, a cut-off below 70 μg/ml for PG1 in the serum, as well as a cut-off below 3.0 for the PG1/2-ratio was defined as pathological. Patients with positive pepsinogen serum test showed a higher prevalence of glandular atrophy (*p *= 0.003 and 0.022, respectively). Differences in further parameters did not show statistical significance. Please see table 4 for details.

**Table 4:** Subgroup characteristics of patients with pathological pepsinogen test

		PG1 < 70 μg/ml			PG1/2-ratio < 3.0		
		**Positive (n = 47)**	**Negative (n = 71)**	***p*-value**	**Positive (n = 20)**	**Negative (n = 98)**	***p*-value**

Laurén type	Intestinal	28 (59.6%)	31 (43.7%)	n.s.	14 (70%)	45 (45.9%)	n.s.
	Diffuse	19 (40.4%)	40 (56.3%)		6 (30%)	53 (54.1%)	

Localisation	Proximal	19 (40.4%)	34 (47.9%)	n.s.	11 (55%)	42 (42.9%)	n.s.
	Distal	28 (59.6%)	37 (52.1%)		9 (45%)	56 (57.1%)	

*H. pylori*	(positive)	40 (85.1%)	59 (83.1%)	n.s.	17 (85%)	82 (83.7%)	n.s.
CagA	(positive)	34 (72.3%)	43 (60.6%)	n.s.	15 (75%)	62 (63.3%)	n.s.

IM	(positive)	39 (83.0%)	47 (66.2%)	0.057	16 (80%)	70 (71.4%)	n.s.
Atrophy*	(positive)	25 (53.2%)	18 (25.4%)	0.003	12 (60%)	31 (31.6%)	0.022

Displayed are absolute numbers as well as percentages with respect to the group with the same test result (PG test positive or negative). Significant differences are marked with an asterisk applying a two-sided significance level of *p *< 0.05.

## Discussion

### General aspects

In the present study, we confirmed that gastric mucosal atrophy and a positive *H. pylori *CagA status are the determinant factors for low pepsinogen levels of patients with gastric cancer. The pepsinogen test for mucosal atrophy is not valid in patients with diffuse type gastric cancer.

The low incidence of glandular atrophy in our population is mainly due to the strict diagnostic criteria as mentioned in the methods section and is in line with data in the literature [21]. It has to be mentioned that we preferred applying the updated Sydney classification over more recently suggested staging systems for gastric mucosal alterations like OLGA or OLGIM [22,23]. The latter systems are still under evaluation in clinical practice and the Sydney-scaled assessment is widely accepted.

### Serum pepsinogens and mucosal changes

Our data confirm the general association between gastric atrophy and decreased PG1 levels in the serum as well as a decreased PG1/2-ratio [13]. The same is seen in case of IM in the stomach which can partly be explained by the strong association between the presence of IM and atrophy. The consistence of our data is confirmed by the inverse correlation between the degree of both atrophy and IM and the serum levels of PG1 and the PG1/2-ratio which has been previously reported [24].

Variable cut-off-values for PG1 and the PG-ratio were applied in former studies [25], but levels below 70 μg/ml and 3.0, respectively, found most acceptance resulting in a sensitivity of 66.7-84.6% and a specificity of 73.5-87.1% for the detection of atrophic gastritis [12,26-28]. Although some authors presented similar values for the detection of GC [27-29], especially the sensitivity for GC screening is significantly lower (36.8%-62.3%) compared to the assessment of gastric atrophy [15,30,31]. In our study, patients with a pathological pepsinogen-test revealed a prevalence of mucosal atrophy that was twice as high as in patients with normal serum pepsinogens.

### Serum pepsinogens and gastric cancer

It has been reported recently, that the results of the PG-test method are independent from the Laurén type [32]. In the present study, only for the PG1/2-ratio a significant difference between intestinal and diffuse type carcinomas was documented which was reported by other authors [7,33]. However, in patients with diffuse type GC pepsinogen values in the serum gave no adequate information about the presence and degree of mucosal atrophy. There was no significant difference neither for PG1 nor for the PG-ratio between patients with and without atrophy. This could be an effect of the low prevalence of IM and atrophy in patients with diffuse type GC. Another explanation might be the diffuse infiltration of teh gastric mucosa by malignant tissue formations.

Serum analysis of PG1, PG2 and G17 can yield information about the distribution of IM and atrophy in the stomach [11,29]. It is still under debate if the location of a gastric adenocarcinoma has a significant influence on the results of these serological tests. Pepsinogen serum values are not different between patients with proximal and distal GC [32,34-38]. But previous studies showing no association to cardia cancer lack of a precise distinction between adenocarcinoma of the distal esophagus and proximal GC with only the latter being associated to chronic atrophic changes [31,39].

According to the updated Sydney-system the degree of glandular atrophy and IM was scaled separately for each antrum and corpus. Each localisation was evaluated separately in addition to the overall evaluation. The results of these analyses were similar so that location dependent bias is excluded in our study.

### Serum pepsinogens and H. pylori

The influence of *H. pylori *infection on serum pepsinogens was mostly evaluated in patients without gastric malignancy. The altered pepsinogen levels were interpreted as reaction to the inflammation process [11,18,40,41]. In the present study, only carriers of the more aggressive CagA-positive strains of *H. pylori *were affected in their PG1-levels. This influence is in line with studies that investigated risk-factor patterns that are associated with more progressive gastric disease [42]. It has been shown, that a positive CagA status is an independent risk factor that increases the *H. pylori *related odds ratio for the development of chronic atrophic gastritis [43,44]. The CagA antigen as major trigger of the inflammatory response might have a further influence on pepsinogen levels at different stages of mucosal transition, even without overt histopathological changes. It has been demonstrated that the CagA status is an individual indicator for atrophic changes in the gastric mucosa independent from pepsinogen levels in the serum [21,30,45,46]. Concerning the effect of *H. pylori *itself on the pepsinogen tests, a statistical bias cannot be excluded since 83.9% of the study population were *H. pylori *positive compared to only 16.1% *H. pylori *negatives.

The influence of CagA was confirmed by the univariate ANOVA-test analysing the influence of the pathobiological characteristics on the pepsinogen values. Besides the presence of atrophic changes only a positive CagA status revealed an independent influence on PG1 levels in the serum. Laurén type and location of the tumor have no influence on the serum-pepsinogen tests as reported before [47].

It is notable that in the present study *H. pylori *status was assessed only by serum-based methods. Therefore, not only patients with actual *H. pylori *infection were classified as *H. pylori*-positive but also patients with prior infection. It has been shown in studies on patients with atrophic gastritis that bacteria are cleared during the development of atrophy [48]. Thus, the infection cannot be detected anymore although the related histomorphological changes were initially caused by *H. pylori*-driven inflammation. Inclusion of patients with a "serological scar" of prior infection prevents underestimation of *H. pylori*-associated processes.

### Gastrin 17 and Pepsinogen 2

G17 and absolute PG2 serum values were not influenced by GC-related factors. It has been reported, that PG2 is upregulated inflammation-dependent in case of *H. pylori *induced gastritis, whereas the data about more severe stages of gastric disease are scarce and only reflected by changes in the PG1/2-ratio [11,31,45]. A recent study on a population in Shanghai reported a three-fold increased risk for GC in case of PG2 levels above 6.6 ng/l in the serum [34].

At the stage of gastritis with or without focal atrophic changes, G17-levels in the serum correlate not only with the degree of the mucosal damage but also with their location in the stomach. However, it is questionable if these associations can be transferred to the stage of invasive GC. Hansen *et al. *reported that there was no difference in G17-levels in patients with proximal and distal GC [34].

## Conclusion

We confirmed that in patients with GC there is no independent association of serum levels for PG1, PG2 and G17 with the Laurén type nor with location of the main tumor mass. Serological assessment of PG1 and the PG1/2-ratio provides an adequate report on the severity of atrophic changes in the gastric mucosa in patients with intestinal type cancer. CagA is positively associated with a decrease of serum PG1 and the PG1/2-ratio.

The study confirms that a serological screening for preneoplastic conditions is possible. Therefore, these serological tests could help to identify individuals at high risk for further gastric cancer development even in regions with low incidence of gastric adenocarcinoma. In patients with an abnormally low serum pepsinogen test, upper gastrointestinal endoscopy should be performed and if premalignant conditions are confirmed these patients should be included in an endoscopic surveillance programme. This strategy has been recommended in a recently published consensus report on the managment of precancerous conditions and lesions in the stomach (MAPS) [49].

Serological screening can be offered in combination with other means of prevention like e.g. screening colonoscopy for colorectal cancer prevention. Prospective multicenter trials are necessary for analysis of cost-effectiveness of this strategy.

## Abbreviations

AEG: adenocarcinoma of the esophagogastric junction; CagA: cytotoxic antigen A; G17: gastrin 17; GC: gastric cancer; *H. pylori: Helicobacter pylori*; IM: intestinal metaplasia; OLGA: operative link for gastritis assessment; OLGIM: operative link on gastric intestinal metaplasia assessment; PG1: pepsinogen I; PG2: pepsinogen II; PG-ratio: pepsinogen-ratio.

## Competing interests

The authors declare that they have no competing interests.

## Authors' contributions

JB did the main part of the data collection, performed the statistical analysis and drafted the manuscript. MS contributed to the data collection and helped to draft the manuscript. TW coordinated and performed the work done in the laboratory (serum analysis). DK provided the pathological data on gastric cancer characteristics and the scoring of gastritis according to the updated Sydney classification. PM as principal supervisor helped to design the study and reviewed the draft of the manuscript. All authors read and approved the final manuscript.

## Pre-publication history

The pre-publication history for this paper can be accessed here:

http://www.biomedcentral.com/1471-230X/12/10/prepub

## Supplementary Material

Additional file 1 **Figure s1: PG1 and the PG1/2-ratio according to Laurén type and CagA status**. A) Patients with intestinal type GC presented lower values for both parameters. The difference was significant only for the PG1/2-ratio (*p *= 0.003) not for PG1 (*p *= 0.062). B) Patients with positive CagA status revealed a trend for lower PG1 levels (*p *= 0.058) but no difference in the PG1/2-ratio compared to CagA negative individuals. Comparison was done by the Mann-Whitney U-test, significance for *p *< 0.05. Click here for file

Additional file 2 **Figure s2: PG1 and the PG1/2-ratio according to the presence of atrophy and IM**. A) Patients with glandular atrophy presented lower values for PG1 (*p *< 0.001) and for the PG1/2-ratio (*p *< 0.001). B) Patients with IM presented lower values for PG1 (*p *= 0.02) and for the PG1/2-ratio (*p *= 0.006). Comparison was done by the Mann-Whitney U-test, significance for *p *< 0.05. Click here for file

Additional file 3 **Figure s3: Correlation of PG1 levels and PG-ratio with the degree of glandular atrophy and IM**. A) For both PG1 (*p *< 0.001; *r *= -0.385) and the PG1/2-ratio (*p *= 0.001; *r *= -0.344) there was an inverse association to the degree of glandular atrophy. B) For both PG1 (*p *< 0.001; *r *= -0.351) and the PG1/2-ratio (*p *= 0.002; *r *= -0.285) there was an inverse association to the degree of IM. Analysis was done by Spearman's rank correlation test, significance for *p *< 0.05. Click here for file

Additional file 4 **Figure s4: Correlation of patients' age with the degree of mucosal changes**. Correlation of patients' age with A) atrophy (*p *= 0.003; *r *= 0.341) and B) with IM (*p *< 0.001; *r *= 0.272). Analysis was done by Spearman's rank correlation test (*p *< 0.05). Click here for file
