# Supplementary figures and images for: Serological assessment of gastric mucosal atrophy in gastric cancer
Source: BMC Gastroenterol. 2012 Jan 31;12:10. doi: 10.1186/1471-230X-12-10 (PMC3280182; doi:10.1186/1471-230X-12-10)

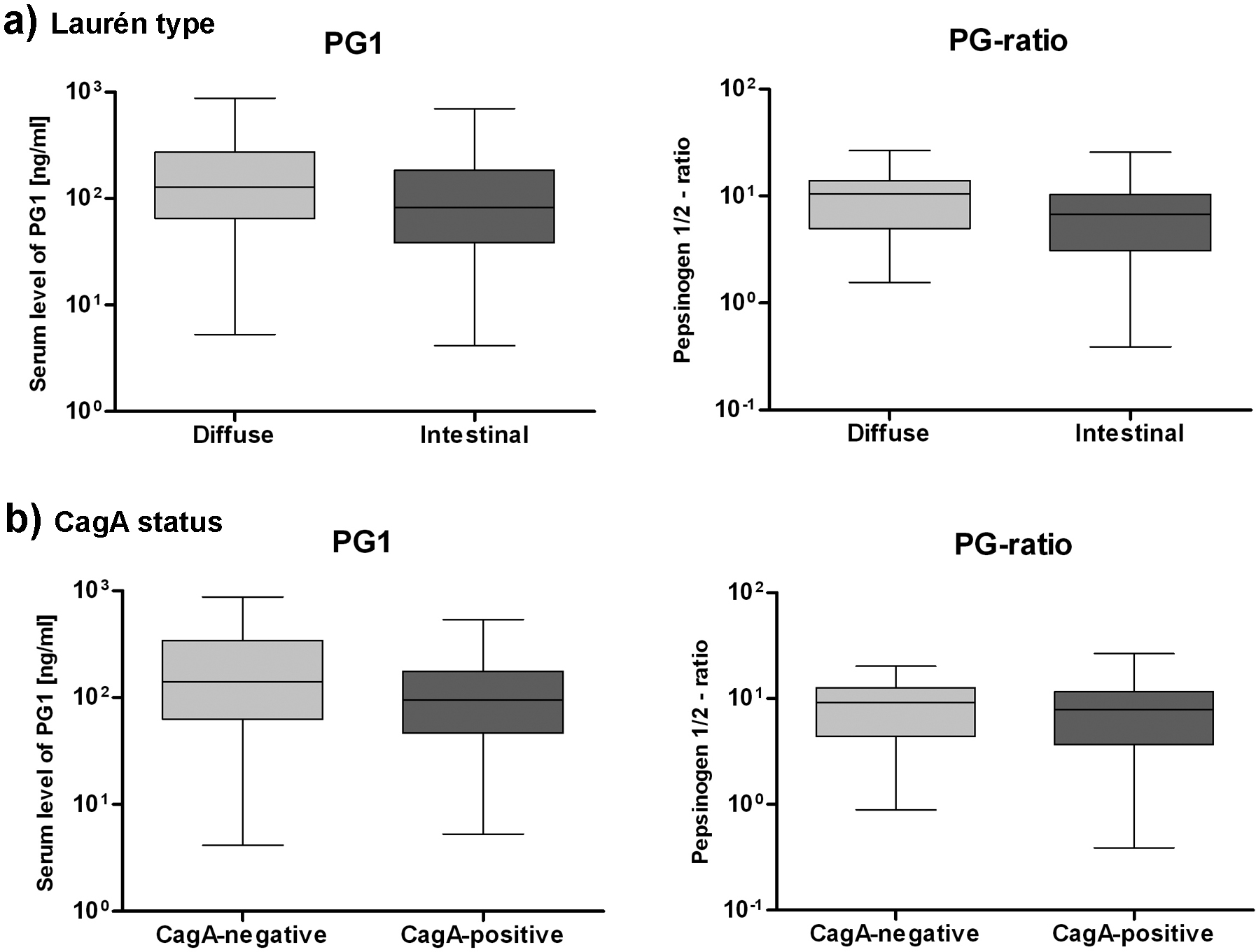

Supplement: Additional file 1 — Figure s1: PG1 and the PG1/2-ratio according to Laurén type and CagA status. A) Patients with intestinal type GC presented lower values for both parameters. The difference was significant only for the PG1/2-ratio (p = 0.003) not for PG1 (p = 0.062). B) Patients with positive CagA status revealed a trend for lower PG1 levels (p = 0.058) but no difference in the PG1/2-ratio compared to CagA negative individuals. Comparison was done by the Mann-Whitney U-test, significance for p < 0.05. [file 1471-230X-12-10-S1.JPEG]

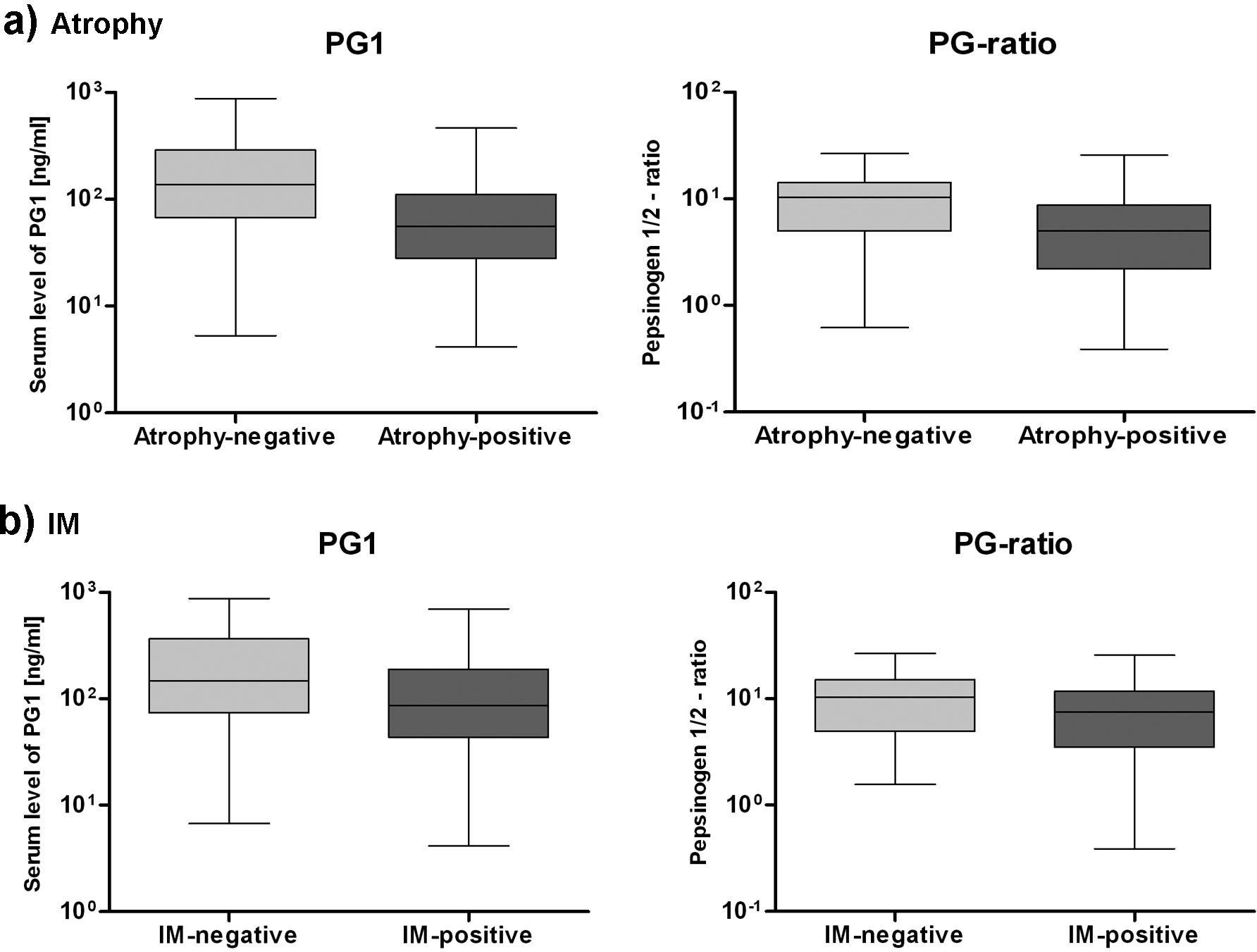

Supplement: Additional file 2 — Figure s2: PG1 and the PG1/2-ratio according to the presence of atrophy and IM. A) Patients with glandular atrophy presented lower values for PG1 (p < 0.001) and for the PG1/2-ratio (p < 0.001). B) Patients with IM presented lower values for PG1 (p = 0.02) and for the PG1/2-ratio (p = 0.006). Comparison was done by the Mann-Whitney U-test, significance for p < 0.05. [file 1471-230X-12-10-S2.JPEG]

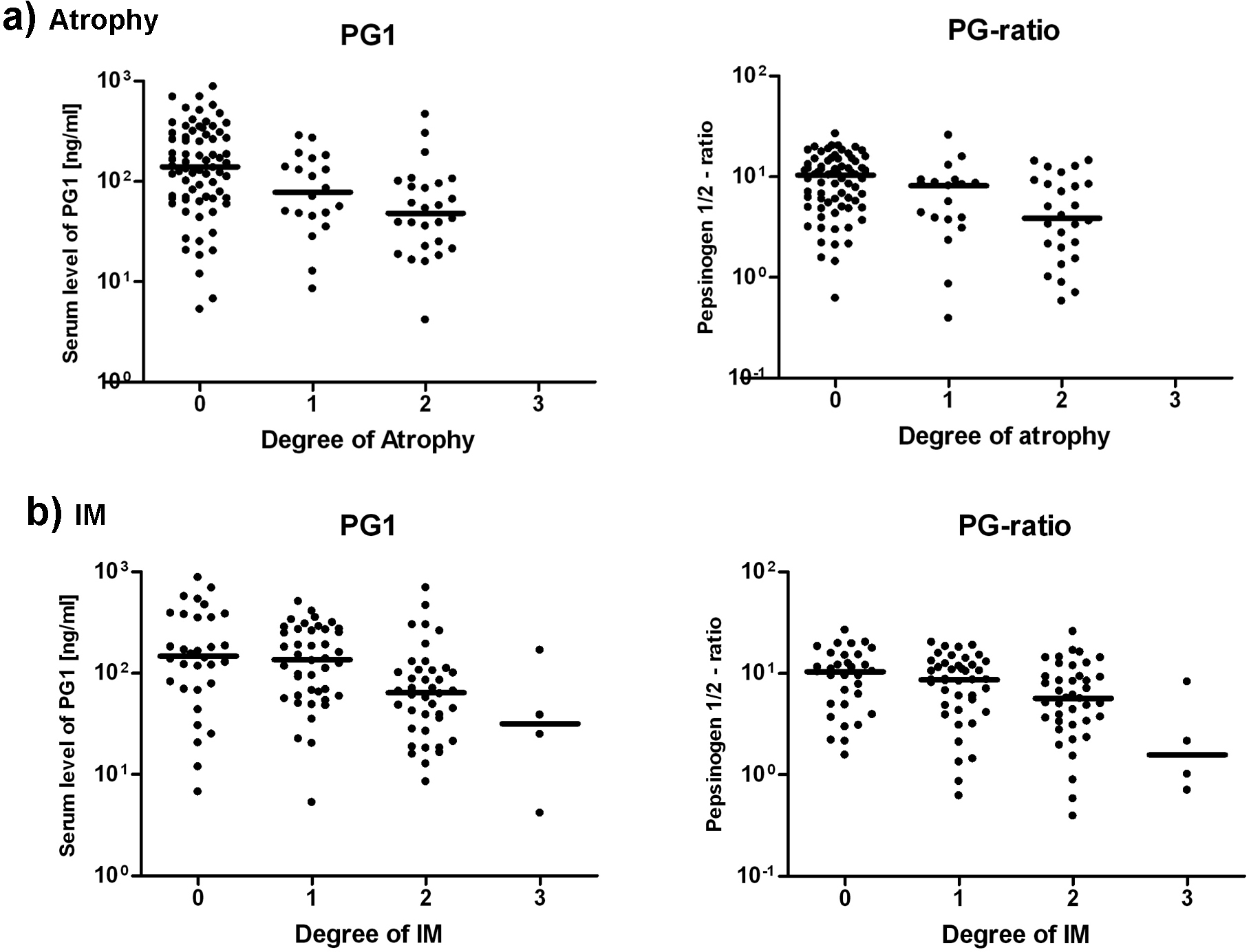

Supplement: Additional file 3 — Figure s3: Correlation of PG1 levels and PG-ratio with the degree of glandular atrophy and IM. A) For both PG1 (p < 0.001; r = -0.385) and the PG1/2-ratio (p = 0.001; r = -0.344) there was an inverse association to the degree of glandular atrophy. B) For both PG1 (p < 0.001; r = -0.351) and the PG1/2-ratio (p = 0.002; r = -0.285) there was an inverse association to the degree of IM. Analysis was done by Spearman's rank correlation test, significance for p < 0.05. [file 1471-230X-12-10-S3.JPEG]

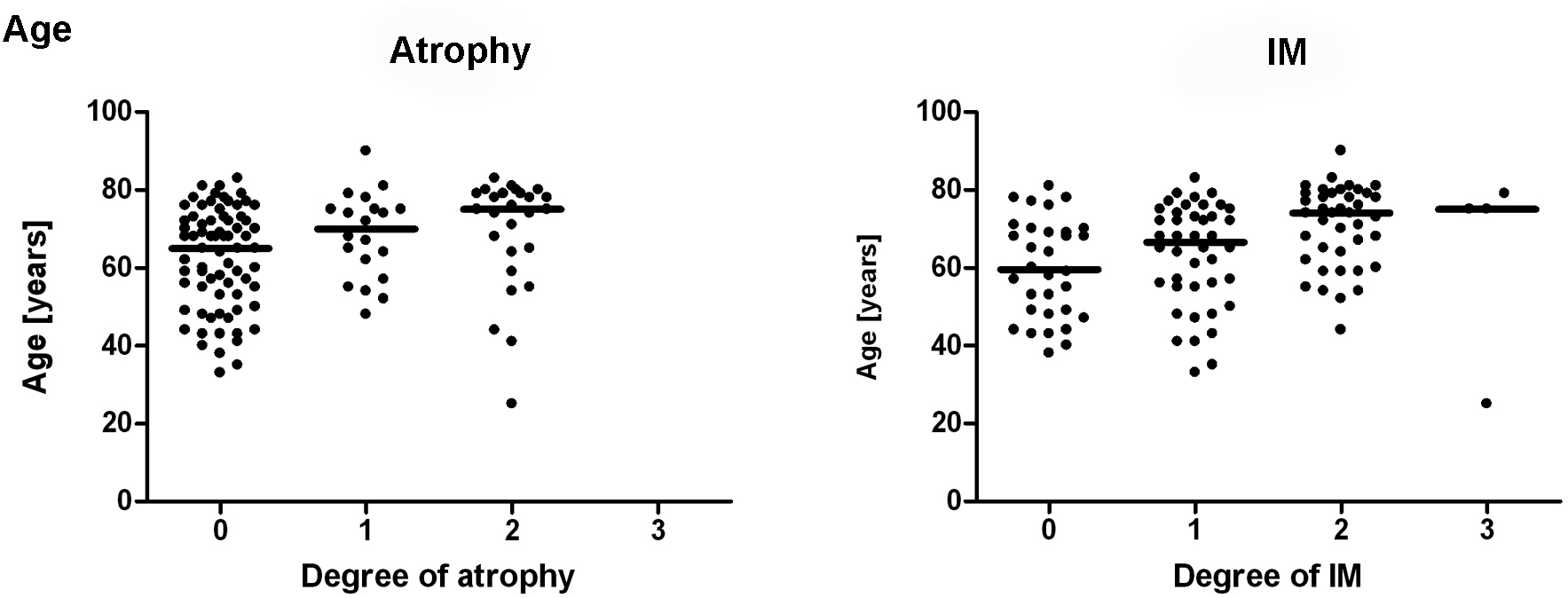

Supplement: Additional file 4 — Figure s4: Correlation of patients' age with the degree of mucosal changes. Correlation of patients' age with A) atrophy (p = 0.003; r = 0.341) and B) with IM (p < 0.001; r = 0.272). Analysis was done by Spearman's rank correlation test (p < 0.05). [file 1471-230X-12-10-S4.JPEG]
